# On the Psychological Barriers to the Workplace: When and Why Metastereotyping Undermines Employability Beliefs of Women and Ethnic Minorities

**DOI:** 10.1037/a0037645

**Published:** 2014-10

**Authors:** Chuma Kevin Owuamalam, Hanna Zagefka

**Affiliations:** 1 School of Psychology, University of Nottingham; 2 Royal Holloway, University of London

**Keywords:** cultural diversity, employability belief and workplace access, self-esteem, social identity, stigma

## Abstract

We investigated the effect of how one might expect one’s group to be viewed by a dominant outgroup (i.e., metastereotypes) on employability beliefs of members of disadvantaged groups. Based on the extensive literature on stereotype threat, we hypothesized that activating negative metastereotypes would undermine employability beliefs of members of disadvantaged groups, because such beliefs are likely to threaten their state self-esteem. In particular, we expected that an effect of negative metastereotyping on employability beliefs would be explained by momentary self-doubts and be particularly evident among members whose dispositional self-esteem is high rather than low to begin with. Taken jointly, results from a correlational study (*n* = 80) and an experimental study (*n* = 56) supported these hypotheses, and discussion focuses on their implications for mobility into the workplace.

The world is currently witnessing a global economic downturn and, not surprising, competition for resources seems more apparent between different social groups in society. In Britain, for example, levels of unemployment are high (Office of National Statistics U.K., 2011) and the situation is worrying for both women and ethnic minorities (Barrett, 2010; Li & Heath, 2010; Office of National Statistics U.K., 2011). It is in this context that we explored the predictors of people’s perceptions of their employment prospects based on the knowledge of their membership of an undervalued social group—a construct we call employability beliefs.

Of particular interest was the impact of negative metastereotyping on employability beliefs. A metastereotype is a belief or an awareness that a relevant outgroup has a certain opinion of one’s own ingroup (Vorauer, Main & O’Connell, 1998; Vorauer, Hunter, Main, & Roy, 2000). Negative metastereotypes have been shown to result in a range of negative consequences. For example, previous evidence shows that an awareness of negative metastereotypes is associated with feeling discriminated against on the basis of one’s gender group among women (Owuamalam & Zagefka, 2013). Corroborating evidence also comes from related research on stereotype threat (Steele, 1992; Steele & Aronson, 1995). Research on stereotype threat generally shows that anxiety over an outgroup’s view of one’s group in a specific domain can negatively impact the performance of members of the stereotyped group in that domain (Steele & Aronson, 1995). It further suggests that members of stigmatized groups can deal with negative ingroup stereotypes by devaluing the stereotyped domain and emphasizing instead those aspects in which their group favorably compares with the outgroup (i.e., disengagement). Members could also deal with stereotype threat by ‘discounting’ the importance of domains in which the ingroup is negatively stereotyped—causing them to view negative outcomes in those domains as a reflection of wider discrimination against the ingroup rather than poor personal ability (Crocker & Major, 1989). They could also remove their self-esteem completely from that domain as a long-term strategy to cope with challenges on the stereotyped domain (so-called dis-identification hypothesis—see Crocker, Major, & Steele, 1998 for a review).

Although stereotype threat continues to receive research attention, work in the area has typically focused on academic and cognitive performance (Chasteen, Bhattacharyya, Horhota, Tam, & Hasher, 2005; see Crocker et al., 1998) as well as physiological well-being (Blascovich, Spencer, Quinn, & Steele, 2001) of members of disadvantaged groups. However, there is little research in comparison on the consequences of negative metastereotyping on work-related attitudes of members of stigmatized groups. The few studies in the area have often examined the impact of an awareness of ingroup stereotypes within organizations and how activating these mind-sets can have negative implications for leadership aspirations (Davies, Spencer, & Steele, 2005) and feedback seeking behaviors of stigmatized individuals (Roberson, Deitch, Brief, & Block, 2003). Others have examined the effect of this mind-set on test performance during an interview—when stigmatized individuals are nearer to the workplace—and have shown that stereotype threat undermines performance at interview (Klein, Pohl, & Ndagijimana, 2007). In short, none of these previous investigations have established that activating negative metastereotypes can have a similar undermining influence when people are *contemplating* whether or not to apply for a job. Although Davies, Spencer, Quinn, and Gerhardstein (2002) have shown that stereotype threatening commercials can increase women’s preference for *specific* vocations in which their gender group is positively valued over the ones in which the ingroup compares unfavorably with men, no other research has articulated the mechanism responsible for this effect or the individuals who may be most vulnerable to it.

Thus, unlike the typical stereotype threat research in which *state* performance anxiety in a *specific* stereotyped domain results in negative outcomes for the stigmatized, in the current study, we sought to examine (a) whether activating negative metastereotypes outside of a *performance* context undermines peoples’ *global* expectations in terms of their employability prospects; (b) the mechanism responsible for this—in the shape of fluctuations in state self-esteem; and (c) how individuals with dispositional high and low self-esteem experience the proposed effects given the traditional emphasis on self-esteem as a coping resource (Baumeister, 2005).

## Metastereotyping and Employability Beliefs

Specifically, we reasoned that because metastereotypes are often negative (Vorauer et al., 1998), are activated when one contemplates intergroup encounters (Vorauer et al., 2000), and engender feelings of rejection (Gordijn & Boven, 2009; see also Mendoza-Denton, Downey, Purdie, Davis, & Pietrzak, 2002); one might then expect metastereotypes to undermine perceived prospects of obtaining employment in a society dominated by the powerful outgroup. Because metastereotypes undermine stigmatized individual’s sense of self-worth (Gordijn & Boven, 2009) we reasoned this negative self-evaluation might be the main reason why, and mechanism through which, negative metastereotypes reduce employability beliefs (see also Branscombe et al., 1999; Schmitt, Branscombe, Kobrynowicz, & Owen, 2002; Dooley & Prause, 1997; Ellis & Taylor, 1983; Gardner & Pierce, 1998).

Of course, people’s attitudes and perceptions of their chances of gaining employment can also depend on the belief that one has got the right qualities to obtain work in the first place. Thus, to the extent that an awareness of negative metastereotypes challenge a positive orientation toward the self, and this momentary downward fluctuation in self-view lowers their expectations of gaining employment, we reasoned that such an effect would be particularly visible among those members whose dispositional self-esteem is high (rather than low) to begin with.

Although those who are dispositionally high in self-esteem are more likely to be approach oriented and have better coping resources than those who are dispositionally low in self-esteem (Cohen & McKay, 1984; see also Baumeister, Campbell, Krueger, & Vohs, 2003; Crocker & Wolfe, 2001) we reasoned this buffer may be applicable only in instances where such members feel able to control events that affect them (Baumeister, 2005; Gordijn & Boven, 2009; Tangney, Baumeister, & Boone, 2004). Negative metastereotypes (outgroup’s opinions of the ingroup) occur largely outside of members’ sphere of control and therefore unlikely that dispositionally high self-esteem group members would feel able to directly control the possibility that a potential job-application would (not) be evaluated via the lens of ingroup stereotypes. That is, people who are dispositionally high in self-esteem may evaluate their employability prospects based not only on an awareness of their personal qualities (controllable events) but also their undervalued social identity (less controllable event). To the extent that members feel confident about their personal qualities, but suspect that their undervalued social identity would pose an obstacle, it seems reasonable that such members would adjust their employability beliefs downward to reflect the pervasive negative regard the larger society holds of ‘people like them’ (Festinger, 1957).

According to social identity theory (Tajfel & Turner, 1986), people’s behavior and attitudes are influenced by their social rather than personal identity when their social group membership is salient, and activating metastereotype is likely to increase the salience of group members’ social identity (Shelton, Richeson, & Vorauer, 2006). Consequently, it is unlikely that those who ordinarily hold high opinions about their personal attributes would have the protection which a sense of their personal qualities affords them. In other words, such group members may hold the suspicion that they would be evaluated by the dominant outgroup on the basis of ingroup stereotypes rather than their personal abilities. Because of this, we expected the undermining effect of metastereotyping on employability beliefs that is explained by state self-esteem to be particularly pronounced among those group members whose dispositional self-esteem is high to begin with.

In contrast, those whose dispositional self-esteem is low to begin with generally hold negative views about their self compared to those whose dispositional self-esteem is high. To the extent that focusing on negative metastereotypes increases the salience of their social identity, it seems likely that such external devaluation would match the pessimistic self-regard of those individuals whose dispositional self-esteem is low. For this reason, we do not expect that employability beliefs of such individuals would visibly fluctuate to the same degree as one might expect among those whose dispositional self-esteem is high.

In sum, activating negative metastereotypes should lower employability beliefs, and this effect should be mediated by deficits in state self-esteem (Hypothesis 1). We also expect this mediated effect to be particularly visible among those whose dispositional self-esteem is high but not low (Hypothesis 2). We tested these predictions in two studies using two historically disadvantaged groups: Women (Study 1) and ethnic minorities (Study 2) in Britain (see Branscombe, 1998; Crosby, 1982; Heath & Cheung, 2007).

## Study 1: Women in Britain

### Method

#### Participants and design

Eighty women were recruited at Keele University campus in the U.K. (*M*_age_ = 20.63, *SD*_age_ = 2.14). At the time of study (2008/9), the ethnic composition of this university was 79.1% white and 20.9% ethnic minority (see Student Equality and Diversity Profile Report, 2010/2011). Metastereotyping (the focal independent variable), dispositional self-esteem (moderator), state self-esteem (mediator), and employability beliefs (outcome variable) were all measured.

#### Materials and procedure

Participants were told the study was interested in attitudes and perceptions that people in British society have about one another. They were asked to complete a measure of dispositional self-esteem using Rosenberg’s 10-item global self-esteem scale: for example, *I feel that I am a person of worth, at least on an equal plane with others*. Responses on this scale were obtained on a 6-point scale (1 = *strongly disagree*, 6 = *strongly agree*; α = .88). High values on this scale indicate high levels of self-esteem.

Next, the negativity of participants’ metastereotype awareness was measured using an item adapted from Owuamalam, Tarrant, Farrow, and Zagefka (2013): *The impressions that men hold about women in this society are generally* . . . (1 = *very negative* to 6 = *very positive*). This scale was reverse coded so that higher scores meant greater negative metastereotyping.

Participants then completed a measure of state self-esteem using the single-item self-esteem scale previously developed and used by Robins, Hendin, and Trzesniewski (2001): *At the moment I have high self-esteem* (1 = *strongly disagree* to 6 = *strongly agree*). Robins et al. (2001) have shown that this item has a strong convergent validity with Rosenberg’s global self-esteem scale.

Finally, participants completed a three-item measure of employability beliefs adapted from Berntson and Marklund (2007). Items were *As a woman, I believe I could get a job in this society without problems*; *Women like me could easily find a job in this society*; and *My identity as a woman makes it easy for me to get a job* (1 = *strongly disagree* to 6 = *strongly agree*; α = .75). They were subsequently debriefed and thanked.

### Results

Table 1 depicts the bivariate correlations among variables in the current study. Of note is the significant negative relationship between metastereotype negativity and employability beliefs: The more negative participants’ metastereotype awareness was the lower their perceived employability prospect.[Table-anchor tbl1]

#### Mediational analysis

To examine the hypothesis that state self-esteem explains the relationship between metastereotyping and employability beliefs, we bootstrapped the confidence interval (CI) around this indirect effect following Shrout and Bolger’s (2002) recommendation. The indirect effect here is the product of the path leading from metastereotyping (the focal predictor) to state self-esteem (the mediator) and the path from this mediator to the outcome (employability beliefs). According to this approach, a mediated effect is achieved if zero lies outside the upper and lower limits of a 95% bootstrapped CI for the estimate of the indirect effect. Confirming Hypothesis 1, results showed that metastereotype negativity lowered employability beliefs partly because of downward fluctuations in state self-esteem (see Table 2).[Table-anchor tbl2]

#### Moderating role of dispositional self-esteem

**Figure 1 fig1:**
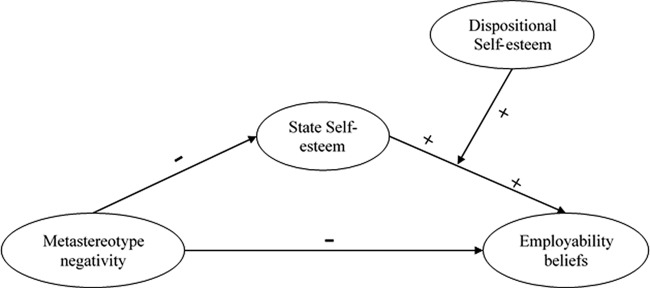
Conceptual model of the effect of negative metastereotyping on employability beliefs via state self-esteem, when dispositional self-esteem is the moderator.

To examine the second hypothesis that the indirect relationship between metastereotype negativity and employability beliefs that was explained by deficits in state self-esteem is mostly evident for those people with high dispositional self-esteem, we computed a conditional indirect effect analysis, by interacting the path from state self-esteem to employability beliefs with dispositional self-esteem (see Figure 1). In this analysis we sought to demonstrate that the *indirect* effect of metastereotype negativity on employability belief was particularly evident for those individuals who are *high* (rather than *low*) in dispositional self-esteem. Following Preacher, Rucker, and Hayes (2007), a conditional indirect effect is established if the upper and lower limits of a bootstrapped 95% CI for the indirect effect of metastereotyping on employability beliefs via state self-esteem at high (+1 *SD* above mean) or low (−1 *SD* below mean) levels of dispositional self-esteem do not contain zero. All predictors were mean centered before analysis (Aiken & West, 1991).[Fig-anchor fig1]

In line with Hypothesis 2, results revealed a negative relationship between metastereotype negativity and employability that is explained by deficits in state self-esteem but only among those women whose dispositional self-esteem was high to begin with and not for those who dispositional self-esteem was low (see Table 3).[Table-anchor tbl3]

### Discussion

Supporting our first hypothesis, we demonstrated that metastereotype negativity was associated with lower employability beliefs, and this was attributable to reduction in state self-esteem. We also showed that this indirect effect was visible only among those group members whose dispositional self-esteem was high (Hypothesis 2). Because this study was correlational, we conducted a follow-up study in which negative metastereotyping was experimentally manipulated so as to enable a causal interpretation of the trends reported in the current study. We also aimed to enhance the generalizability of the patterns shown in this study to other disadvantaged groups by using a different intergroup context to the one reported in the current study.

## Study 2: Ethnic Minorities in Britain

### Method

#### Participants and design

Fifty-six people of South Asian ethnic decent were recruited from Staffordshire University, U.K. (32 men and 24 women; *M*_age_ = 20.32, *SD*_age_ = 1.39; 80.4% reported having British nationality). In terms of ethnic composition, 32.1% self-reported being Indian, 33.9% Pakistani, 24.2% Asian, 5.4% Bangladeshi, 3.6% Malay, and 1.8% Panthani. A between-groups design was used: Metastereotyping (the focal independent variable) was manipulated, while dispositional self-esteem (moderator), state self-esteem (mediator), and employability beliefs (outcome variable) were measured.

#### Materials and procedure

As in Study 1, we measured dispositional self-esteem using Rosenberg’s 10-item global self-esteem scale (1 = *strongly disagree* to 6 = *strongly agree*; α = .87). Next, we manipulated metastereotype valence using the procedure developed by Branscombe (1998; see also Owuamalam & Zagefka, 2011). In one condition participants were instructed to reflect on the negative impressions that native white British people hold of ethnic minorities in this society (negative metastereotype condition), while in another participants were instructed to think about the positive impressions that native British people hold about ethnic minorities (positive metastereotype condition). A third condition was also included in which participants did not receive any metastereotype related instruction (control condition). We did not make any specific predictions regarding the effect of positive metastereotyping and wanted only to explore whether or not it has any potential positive effect on our outcome (compared to the baseline condition)—particularly because such stereotypes pose little (if at all any) concern for group members.

Following the manipulation of metastereotype valence, participants then completed the same state self-esteem measure used in Study 1 (1 = *strongly disagree* to 6 = *strongly agree*). Finally, they completed the same measure of employability beliefs as before, but adapted to reflect the current intergroup context (1 = *strongly disagree* to 6 = *strongly agree*; α = .78). On completion, participants were debriefed and thanked.

### Results

Bivariate correlations and descriptive statistics are presented in Table 1. Unlike in Study 1, the effect of metastereotyping on employability beliefs did not reach statistical significance, *F*_(2, 53)_ = 1.93, *p* = .16, η^2^ = .07, although the pattern was consistent with our expectations (negative metastereotype condition, *M* = 3.90, *SD* = 0.70; control condition, *M* = 4.31, *SD* = 0.98; and positive metastereotype condition, *M* = 4.40, *SD* = 0.86).

#### Mediational analysis

One downside of a causal approach to establishing a mediated effect is the requirement for the relationship between the focal predictor and the outcome to be significant. This is not a requirement in the test of the indirect effect proposed by Preacher et al. (2007; see also Hayes, 2009). Therefore, despite the lack of a significant main effect of metastereotyping on employability belief in the current study, we again bootstrapped the predicted indirect effect. If negative metastereotyping does undermine employability beliefs via its negative effect on state self-esteem, then this effect should be noticeable in the negative metastereotype condition relative to both the positive metastereotype and control conditions. For this reason we dummy coded the effect attributable to negative metastereotyping such that Negative metastereotyping = 2, positive metastereotyping = −1 and control = −1. Further, we explored whether or not positive metastereotyping has any beneficial effect on employability beliefs compared to a control condition in which both valence of the metastereotypes are absent using the following dummy codes: Positive metastereotyping = 1, control = −1, negative metastereotyping = 0 (zero). This coding allows for a contrast between positive metastereotyping and control.

Full model results for the indirect effect of metastereotyping on employability beliefs are presented in Table 2. As expected, group members who activated negative metastereotypes reported reduced employability beliefs and this effect was, again, attributable to a reduction in state self-esteem (95% CI: LL = −.185, UL = .023). Group members’ employability beliefs showed no measureable fluctuation when the comparison was between those who activated positive metastereotypes and those in the control condition (95% CI: LL = −.217, UL = .013). Because of this, we focused subsequent analysis on the effects of negative metastereotyping.

#### Moderating role of dispositional self-esteem

Corroborating the patterns of relationships in Study 1, results from a conditional process analysis showed, in line with expectation, that negative metastereotyping lowered employability beliefs via state self-esteem (see Table 2): but again, only for those group members whose dispositional self-esteem was high rather than low (see Table 3).

### Discussion

Experimental evidence from the current study clearly corroborates the patterns we showed in Study 1. Importantly, both studies provide clear support for the two central predictions: Revealing that activating negative metastereotypes undermines employability beliefs via their negative effect on state self-esteem, and only for members whose dispositional self-esteem was high.

## General Discussion

We set out to test three propositions: (a) that activating negative metastereotypes would undermine employability beliefs of members of stigmatized groups, (b) that such an undermining effect may be explained by momentary self-doubts arising from external regard for one’s social groups, and (c) that people who are high (rather than low) in dispositional self-esteem would experience the proposed undermining effect more. Concerning the first proposition, results revealed a negative relationship between negative metastereotyping and employability beliefs as predicted (Study 1). Although this effect was not significant in Study 2, the pattern of the relationship was similar to that reported in Study 1.

Second, although the finding that negative metastereotyping lowers state self-view is not new (e.g., Gordijn & Boven, 2009; Owuamalam & Zagefka, 2011), the current findings are novel because they show that experiencing momentary self-doubts following concerns over the ways in which one’s group is stigmatized by a more powerful outgroup can significantly undermine stereotyped individuals’ attitudes toward employment. In that respect, then, our findings extend previous research on workplace consequences of social stigma, by examining the mind-sets that could obstruct stigmatized individuals’ chances of gaining access into the workplace in the first place (cf. Klein et al., 2007). After all, attitudes toward seeking employment can have serious implications for actual job-seeking behavior (Ajzen & Fishbein, 2005; Davies et al., 2002).

Of course this is not to say that activating negative metastereotypes will *always* result in reduced inclination, or complete apathy toward job-seeking. Rather the current data suggest that group members may lower their expectations—presumably to reduce the extent to which they experience uncomfortable feelings of disappointment in the aftermath of an unsuccessful job-seeking endeavor. It is unlikely that stereotyped group members in the current study are simply disengaging or dis-identifying with employment, especially because the observed trend is only apparent for those group members who would ordinarily hold optimistic views about their prospects (i.e., those whose dispositional self-esteem is high). These suggestions, however, remain largely untested and therefore could form the basis of future investigation.

The finding that activating negative metastereotypes undermines employability beliefs via its adverse effects on state self-esteem of those who are stereotyped is notable for another reason: Rather than the traditional focus on *specific* domains in which the ingroup compares unfavorably with a relevant outgroup, it suggests that within the context of work, concerns over negative metastereotype could have generic potency even outside work domains in which one’s group is negatively valued (cf. Davies et al., 2002). Thus, in conjunction with evidence from the stereotype threat literature (Davies et al., 2002), future studies could examine, for example, whether or not lowered employability beliefs actually reduces resilience at job-seeking, even on those domains in which the ingroup is positively valued. This is especially important in relation to ethnic migrants, because anti-migrant sentiments among the mainstream may enhance pessimisms over their job prospects in domains in which the ingroup is positively valued (e.g., anti-eastern European sentiments in Britain could discourage Polish workers from seeking construction work in the U.K.—a domain in which the Poles are traditionally positively stereotyped, cf. Winder, 2005).

Furthermore, and although the indirect effect estimates for the two different groups (women and ethnic minorities) across the two studies were significant, the magnitude of this estimate among the ethnic minority sample was more than twice that of women, even with a relatively modest sample size. This suggests that the pattern we present here may be pronounced among members of ethnic minority groups, particularly in the current economic downturn when interethnic tensions and anti-immigration sentiments directed toward ethnic minorities are on the rise (such as from the English Defense League [EDL] and the British National Party [BNP]; see also Winder, 2005).

### Limitations

One limitation of the current study is our use of the single-item measure of self-esteem. Although this measure was previously developed and used by Robins et al. (2001), and has been shown by these authors to have convergent validity with the Rosenberg’s (1979) global self-esteem scale, questions remain about its reliability. Having said that, the positive relationship between this single-item self-esteem scale and Rosenberg’s global self-esteem scale is comparable with those reported in Robins et al. (2001). We also observed identical patterns of relationships between this single-item state self-esteem measure and employability beliefs to those reported for Rosenberg’s global self-esteem across the two studies—demonstrating a similar convergent validity of this scale. The fact that it yielded identical outcomes across two intergroup contexts lends credibility to it. However, and although some methodologists have demonstrated that multiple-item scales show little comparative advantage over their single-item counterparts (Gardner, Cummings, Dunham & Pierce, 1998), future research could benefit from a more rigorous measurement of this construct, for example, by including different multiple-item measures of state self-esteem.

### Conclusion

We showed in two studies that members of stigmatized groups who activate negative metastereotypes downgrade their employability prospects in a society dominated by their privileged outgroup counterparts, and that this was because ingroup stigma reduced their self-worth. Contrary to extant perspectives on self-esteem effects, we showed that it was people with high (but not those with low) dispositional self-esteem that were particularly vulnerable to these effects. For charities and social welfare professionals who work with disadvantaged communities, the current findings provide novel insights into an important process that could underlie resilience at job-seeking. It further identifies those individuals who may be thought to have the resources to cope with stigma (i.e., people with high self-esteem) and therefore may ordinary escape the radar of support, as those particularly likely to benefit from support.

## Figures and Tables

**Table 1 tbl1:** Bivariate Correlations (and Descriptive Statistics) of Variables in Studies 1 and 2

Variable	1	2	3	4	*M*	*SD*
1. Dispositional self-esteem	1	−0.24*	0.64**	.21^†^	4.37 (5.00)	0.80 (0.69)
2. Metastereotype negativity	−0.09 (−0.15)	1	−0.22*	−0.27*	3.11 (—)	0.86 (—)
3. State self-esteem	.48**	−0.40** (−0.17)	1	0.31**	4.01 (4.80)	1.15 (0.98)
4. Employability beliefs	.31*	−0.26^†^ (0.05)	0.39**	1	4.31 (4.20)	0.88 (0.86)
*Note*. Study 1 correlation coefficients are shown in the upper diagonal of the correlation matrix, whereas that of Study 2 are shown below the diagonal. Study 1 means (*M*) and standard deviation (*SD*) are presented outside the parentheses, whereas those for Study 2 are presented in parentheses. In Study 2, correlation coefficients for negative metastereotyping (coded: negative 2, control −1, and positive −1) are reported outside the parenthesis while those for positive metastereotyping (coded: positive 1, control −1, negative 0) are reported within brackets.						
^†^ *p* < .10. * *p* ≤ .05. ** *p* ≤ .01, respectively (all two-tailed).						

**Table 2 tbl2:** Indirect Effects of Metastereotyping on Employability Beliefs via State Self-Esteem

Variable		Study 1: Women				Study 2: Ethnic minorities		
		*B*	*SE*		Two-tailed *p*	*B*	*SE*	Two-tailed *p*
Mediator variable model								
Metastereotyping (MS)		−0.30	0.15		0.05	−0.25 (−0.22)	0.09 (0.15)	0.01 (0.15)
Dependent variable model								
Total effect of MS		−0.28	0.11		0.02	−0.15 (0.04)	0.08 (0.14)	0.06 (0.76)
Direct effect of MS		−0.22	0.11		0.05	−0.07 (0.12)	0.08 (0.14)	0.39 (0.39)
Direct effect of State self-esteem (SSE)		0.20	0.08		0.02	0.33	0.12	0.01
			95% CI				95% CI	
	Boot *B*	Boot *SE*	LL	UL	Boot *B*	Boot *SE*	LL	UL
Indirect effect of MS via SSE	−0.06	0.04	−0.163	−0.009	−0.08 (−0.07)	0.04 (0.06)	−0.186 (−0.211)	−0.019 (0.016)
*Note*. Number of bootstrap resamples 5,000 (*n* size: Study 1 = 80, Study 2 = 56). In Study 2, effects attributable to negative metastereotyping (coded: negative 2, control −1, and positive −1) are reported outside the parenthesis, whereas effects attributable to positive metastereotyping (coded: positive 1, control −1, negative 0) are reported within brackets. *B* = Unstandardized beta weights, *SE* = standard error. Boot *B* = bootstrapped indirect effect estimate; Boot *SE* = bootstrapped standard error for the indirect effect estimate; Bias corrected and accelerated CI is reported here to adjust for possible bias (i.e., the difference between estimate from the original sample and its bootstrapped equivalent) and skewness in the bootstrap distribution (Efron, 1987).								

**Table 3 tbl3:** The Conditional Indirect Effect of Negative Meta-Stereotyping on Employability Beliefs via State Self-Esteem When Dispositional Self-Esteem Is the Moderator

Variable		Study 1: Women				Study 2: Ethnic minorities		
		*B*	*SE*		Two-tailed *p*	*B*	*SE*	Two-tailed *p*
Mediator variable model (state self-esteem)								
Metastereotyping (MS)		−0.30	0.15		0.05	−0.25 (−0.22)	0.09 (0.15)	0.01 (0.15)
Dependent variable model (employability beliefs)								
MS		−0.16	0.11		0.16	−0.06 (0.12)	0.08 (0.13)	0.49 (0.37)
Dispositional self-esteem (DSE)		0.05	0.15		0.73	0.47	0.21	0.03
State self-esteem (SSE)		0.28	0.11		0.01	0.27	0.13	0.04
SSE × DSE		0.17	0.07		0.03	0.34	0.15	0.03
Conditional indirect effect of MS on employability beliefs via SSE at ± 1SD of mean DSE			95% CI				95% CI	
	Boot *B*	Boot *SE*	LL	UL	Boot *B*	Boot *SE*	LL	UL
High dispositional self-esteem [+1 *SD*]	−0.12	0.07	−0.303	−0.028	−0.13 (−0.11)	0.06 (0.08)	−0.274 (−0.318)	−0.031 (0.015)
Low dispositional self-esteem [−1 *SD*]	−0.04	0.04	−0.145	0.021	−0.01 (−0.01)	0.05 (0.05)	−0.108 (−0.146)	0.086 (0.056)
*Note*. Number of bootstrap resamples = 5000 (observed *n*s = 80 [Study 1], 56 [Study 2]). *B* = Unstandardized beta weights, *SE* = standard error. Boot *B* = bootstrapped indirect effect estimate; Boot *SE* = bootstrapped standard error for the indirect effect estimate; Bias corrected and accelerated CIs are reported. In Study 2, effects attributable to negative metastereotyping (coded: negative 2, control −1, and positive −1) are reported outside the parenthesis, whereas effects attributable to positive metastereotyping (coded: positive 1, control −1, negative 0) are reported within brackets.								

## References

[c1] Aiken L. S., & West S. G. (1991). Multiple regression: Testing and interpreting interactions. Newbury Park, CA: Sage

[c2] Ajzen I., & Fishbein M. (2005). The influence of attitudes on behavior In Albarracín D., Johnson B. T., & Zanna M. P. (Eds.), The handbook of attitudes (pp. 173–221). Mahwah, NJ: Erlbaum

[c4] Barrett R. (2010). Disadvantaged groups in the labour market. Economic and Labour Market Review, 4, 18. doi:10.1057/elmr.2010.78

[c58] Baumeister R. (2005). Rethinking self-esteem: Why nonprofits should stop pushing self-esteem and start endorsing self-control. Stanford Social Innovation Review, (Winter), 34–41

[c6] Baumeister R. F., Campbell J. D., Krueger J. I., & Vohs K. D. (2003). Does high self-esteem cause better performance, interpersonal success, happiness, or healthier lifestyles? Psychological Science in the Public Interest, 4, 1–44. doi:10.1111/1529-1006.01431 26151640

[c7] Berntson E., & Marklund S. (2007). The relationship between perceived employability and subsequent health. Work & Stress, 21, 279–292. doi:10.1080/02678370701659215

[c8] Blascovich J., Spencer S. J., Quinn D., & Steele C. (2001). African Americans and high blood pressure: The role of stereotype threat. Psychological Science, 12, 225–229. doi:10.1111/1467-9280.00340 11437305

[c9] Branscombe N. R. (1998). Thinking about one’s gender group’s privileges or disadvantages: Consequences for well-being in women and men. British Journal of Social Psychology, 37, 167–184. doi:10.1111/j.2044-8309.1998.tb01163.x 9639862

[c10] Branscombe N. R., Schmitt M. T., & Harvey R. D. (1999). Perceiving pervasive discrimination among African-Americans: Implications for group identification and well- being. Journal of Personality and Social Psychology, 77, 135–149. doi:10.1037/0022-3514.77.1.135

[c11] Chasteen A. L., Bhattacharyya S., Horhota M., Tam R., & Hasher L. (2005). How feelings of stereotype threat influence older adults’ memory performance. Experimental Aging Research, 31, 235–260. doi:10.1080/03610730590948177 16036721 PMC1751474

[c59] Cohen S., & McKay G. (1984). Social support, stress, and the buffering hypothesis: A theoretical analysis In Baum A., Singer J. E., & Taylor S. E. (Eds.), Handbook of psychology and health, vol. 4 Hillsdale, NJ: Erlbaum

[c60] Crocker J., & Major B. (1989). Social stigma and self-esteem: The self protective properties of stigma. Psychological Review, 96, 608–630

[c61] Crocker J., Major B., & Steele C. (1998). Social stigma In Gilbert D., Fiske S., & Lindzey G. (Eds.), The handbook of social psychology (4th ed., Vol. 2, pp. 504–553). Boston: McGraw-Hill

[c13] Crocker J., & Wolfe C. T. (2001). Contingencies of self-worth. Psychological Review, 108, 593–623. doi:10.1037/0033-295X.108.3.593 11488379

[c14] Crosby F. J. (1982). Relative deprivation and working women. New York, NY: Oxford University Press

[c15] Davies P. G., Spencer S. J., Quinn D. M., & Gerhardstein R. (2002). Consuming images: How television commercials that elicit stereotype threat can restrain women academically and professionally. Personality and Social Psychology Bulletin, 28, 1615–1628. doi:10.1177/014616702237644

[c16] Davies P. G., Spencer S. J., & Steele C. M. (2005). Clearing the air: Identity safety moderates the effects of stereotype threat on women’s leadership aspirations. Journal of Personality and Social Psychology, 88, 276–287. doi:10.1037/0022-3514.88.2.276 15841859

[c18] Dooley D., & Prause J. (1997). School-leavers’ self-esteem and unemployment: Turning point or a station on a trajectory? In Gotlib I. H. & Wheaton B. (Eds.), Trajectories and turning points: Stress and adversity over the life course (pp. 91–113). New York, NY: Cambridge University Press. doi:10.1017/CBO9780511527623.005

[c19] Efron B. (1987). Better bootstrap confidence intervals. Journal of the American Statistical Association, 82, 171–185. doi:10.1080/01621459.1987.10478410

[c20] Ellis R., & Taylor M. (1983). Role of self-esteem within the job search process. Journal of Applied Psychology, 68, 632–640. doi:10.1037/0021-9010.68.4.632

[c22] Festinger L. (1957). A theory of cognitive dissonance. Evanston, IL: Row Peterson

[c23] Gardner D. G., Cummings L. L., Dunham R., & Pierce J. L. (1998). Single-item versus multiple-item measurement scales: An empirical comparison. Educational and Psychological Measurement, 58, 898–915. doi:10.1177/0013164498058006003

[c24] Gardner D. G., & Pierce J. L. (1998). Self-esteem and self-efficacy within the organizational context: An empirical examination. Group & Organization Management, 23, 48–70. doi:10.1177/1059601198231004

[c26] Gordijn E. H., & Boven G. (2009). Loneliness among people with HIV in relation to locus of control and negative meta-stereotyping. Basic and Applied Social Psychology, 31, 109–116. doi:10.1080/01973530902880266

[c27] Hayes A. F. (2009). Beyond Baron and Kenny: Statistical mediation analysis in the new millennium. Communication Monographs, 76, 408–420. doi:10.1080/03637750903310360

[c29] Heath A. F., & Cheung S. Y. (2007). Unequal chances: Ethnic minorities in western labour markets. Oxford, UK: Oxford University Press

[c30] Klein O., Pohl S., & Ndagijimana C. (2007). The influence of intergroup comparisons on Africans’ intelligence performance in a job selection context. The Journal of Psychology: Interdisciplinary and Applied, 141, 453–468. doi:10.3200/JRLP.141.5.453-468 17933401

[c32] Li Y., & Heath A. (2010). Struggling onto the ladder, climbing the rungs: Employment status and class position by minority ethnic groups in Britain (1972–2005). In Stillwell J., Norman P., Thomas C., & Surridge P. (Eds.) Population, employment, health and well-being (pp. 83–97). New York, NY: Springer

[c33] Mendoza-Denton R., Downey G., Purdie V. J., Davis A., & Pietrzak J. (2002). Sensitivity to status-based rejection: Implications for African American students’ college experience. Journal of Personality and Social Psychology, 83, 896–918. doi:10.1037/0022-3514.83.4.896 12374443

[c35] Office of National Statistics, U. K (2011). Labour market statistics [from December 2010 to February 2011]. Statistical Bulletin. Retrieved May 25, 2011, from http://www.statistics.gov.uk/pdfdir/lmsuk0211.pdf

[c36] Owuamalam C. K., Tarrant M., Farrow C. V., & Zagefka H. (2013). The effect of metastereotyping on judgments of higher-status outgroups when reciprocity and social image improvement motives collide. Canadian Journal of Behavioural Science/Revue canadienne des sciences du comportement, 45, 12–23

[c37] Owuamalam C. K., & Zagefka H. (2011). Downplaying a compromised social image: The effect of metastereotype valence on social identification. European Journal of Social Psychology, 41, 528–537. doi:10.1002/ejsp.805

[c38] Owuamalam C. K., & Zagefka H. (2013). We’ll never get past the glass-ceiling! Metastereotyping, world-views and perceived relative group-worth. British Journal of Psychology, 104, 543–562 24094283 10.1111/bjop.12006

[c40] Preacher K. J., Rucker D. D., & Hayes A. F. (2007). Addressing moderated mediation hypotheses: Theory, methods, and prescriptions. Multivariate Behavioral Research, 42, 185–227. doi:10.1080/00273170701341316 26821081

[c41] Roberson L., Deitch E. A., Brief A. P., & Block C. J. (2003). Stereotype threat and feedback seeking in the workplace. Journal of Vocational Behavior, 62, 176–188. doi:10.1016/S0001-8791(02)00056-8

[c42] Robins R. W., Hendin H. M., & Trzesniewski K. H. (2001). Measuring global self-esteem: Construct validation of a single-item measure and the Rosenberg self-esteem scale. Personality and Social Psychology Bulletin, 27, 151–161. doi:10.1177/0146167201272002

[c44] Rosenberg M. (1979). Conceiving the self. New York, NY: Basic Books

[c45] Schmitt M. T., Branscombe N. R., Kobrynowicz D., & Owen S. (2002). Perceiving discrimination against one’s gender group has different implications for well-being in women and men. Personality and Social Psychology Bulletin, 28, 197–210. doi:10.1177/0146167202282006

[c47] Shelton J. N., Richeson J. A., & Vorauer J. D. (2006). Threatened identities and interethnic interactions. European Review of Social Psychology, 17, 321–358. doi:10.1080/10463280601095240

[c48] Shrout P. E., & Bolger N. (2002). Mediation in experimental and non-experimental studies: New procedures and recommendations. Psychological Methods, 7, 422–445. doi:10.1037/1082-989X.7.4.422 12530702

[c51] Steele C. M. (1992). Race and the schooling of African Americans. Atlantic, 269, 68–78

[c52] Steele C. M., & Aronson J. (1995). Stereotype threat and the intellectual test performance of African Americans. Journal of Personality and Social Psychology, 69, 797–811. doi:10.1037/0022-3514.69.5.797 7473032

[c53] Student Equality and Diversity Profile Report. (2010/11). Retrieved February 25, 2014, from http://www.keele.ac.uk/media/keeleuniversity/equaldiversity/STUDENT%20EQUALITY%20AND%20DIVERSITY%20PROFILE%20REPORT%202011%20-%2012.pdf

[c54] Tajfel H., & Turner J. C. (1986). The social identity theory of intergroup behaviour In Worchel S. and Austin W. G. (Eds.), Psychology of intergroup relations (pp. 7–24). Chicago, IL: Nelson Hall

[c62] Tangney J. P., Baumeister R. F., & Boone A. L. (2004). High self-control predicts good adjustment, less pathology, better grades, and interpersonal success. Journal of Personality, 72, 271–324 15016066 10.1111/j.0022-3506.2004.00263.x

[c55] Vorauer J. D., Hunter A. J., Main K. J., & Roy S. A. (2000). Meta-stereotype activation: Evidence from indirect measures for specific evaluative concerns experienced by members of dominant groups in intergroup interaction. Journal of Personality and Social Psychology, 78, 690–707. doi:10.1037/0022-3514.78.4.690 10794374

[c56] Vorauer J. D., Main K. J., & O’Connell G. B. (1998). How do individuals expect to be viewed by members of lower status groups? Content and implications of meta-stereotypes. Journal of Personality and Social Psychology, 75, 917–937. doi:10.1037/0022-3514.75.4.917 9825528

[c57] Winder R. (2005). Bloody foreigners. London, UK: Abacus

